# Axonemal microtubule dynamics in the assembly and disassembly of cilia

**DOI:** 10.1042/BST20240688

**Published:** 2025-01-31

**Authors:** Yi Zhang, Mu He, Junmin Pan

**Affiliations:** 1 MOE Key Laboratory of Protein Sciences，State Key Laboratory of Complex, Severe, and Rare Diseases, Tsinghua-Peking Center for Life Sciences, School of Life Sciences, Tsinghua University, Beijing 100084, China; 2 Laboratory for Marine Biology and Biotechnology, Qingdao Marine Science and Technology Center, Qingdao, China; 3 School of Biomedical Sciences, The University of Hong Kong, Hongkong, China

**Keywords:** Cilia and flagella, axonemal microtubules, ciliogenesis, kinesin, microtubule-binding proteins

## Abstract

Cilia and eukaryotic flagella (exchangeable terms) function in cell motility and signaling, which are pivotal for development and physiology. Cilia dysfunction can lead to ciliopathies. Cilia are usually assembled in quiescent and/or differentiated cells and undergo disassembly when cells enter cell cycle or in response to environmental stresses. Cilia contain a microtubule-based structure termed axoneme that comprises nine outer doublet microtubules with or without a pair of central microtubules, which is ensheathed by the ciliary membrane. Regulation of the axonemal microtubule dynamics is tightly associated with ciliary assembly and disassembly. In this short review, we discuss recent findings on the regulation of axonemal microtubules by microtubule-binding proteins and microtubule modulating kinesins during ciliary assembly and disassembly.

## Introduction

Cilia and eukaryotic flagella are conserved microtubule-based organelles, present in organisms ranging from unicellular eukaryotes to humans. Their functions in cell motility and signaling are essential for organismal development and physiology. Ciliary dysfunctions are linked to a cohort of human diseases and/or developmental disorders, termed ciliopathies [1,2].

Cilia are assembled from the mother centrioles for primary cilia and duplicated centrioles for multiple motile cilia [3,4]. Docking of cellular membranes to the distal appendages of the mother centriole triggers the removal of centriolar proteins CP110 and CEP97, which prevent microtubule (MT) elongation from the triplet MTs of the centriole. The transition zone is then formed in which nine doublet MTs assemble from the A- and B-tubules of the triplet microtubules of the centriole, followed by further MT extension to form the axonemal MTs [5]. For motile cilia, a pair of central MTs are assembled at the distal end of the transition zone along with the doublet MTs [6]. Upon entering the cell cycle, during cell differentiation or in response to various environmental changes, cilia may undergo disassembly [7,8].

MTs are dynamic structures that are under control by an array of mechanisms, including the proposed “tubulin code”, which represents a combination of tubulin isotypes and post-translational modifications, as well as MT binding proteins and enzymes that depolymerize or sever MTs [9–11]. Regulation of axonemal MT dynamics is tightly associated with ciliary assembly and disassembly. In this short review, we only discuss recent findings pertinent to MT binding proteins and kinesins that have functions in modulating ciliary MT dynamics. For additional information, the readers are referred to previous reviews [12–15].

## Regulation of axonemal MT dynamics by MT binding proteins

### EB1 and EB3

End-binding proteins (EBs) are MT plus end binding proteins that track and accumulate at the plus end of the growing MTs. In mammals, the EB family has three members including EB1, EB2, and EB3. EB1 and EB3 but not EB2 have been found in cells to promote persistent MT growth by suppressing catastrophes [16]. Interestingly, EBs can promote catastrophes *in vitro*, suggesting that EBs in cells act in concert with other MT regulators to control MT dynamics [16]. EB1 binds an intermediate tubulin hydrolyzing state via its calponin homology domain and acts as a scaffold to recruit a number of MT plus end tracking proteins, which contain CAP-GLY or SxIP motifs [17,18]. In *Chlamydomonas*, EB1 has first been shown to localize to the ciliary tip as well as the basal body. Interestingly, it also associates with the ciliary tip of shortening cilia [19], although the underlying mechanism is not defined. It is unknown whether and how EB1 functions in ciliogenesis of *Chlamydomonas*. In mammalian cells, EB1 and EB3 positively regulate the assembly of primary cilia [20,21]. Both localize to the centrioles and/or basal bodies of primary cilia while EB3 also localizes to the tip of motile cilia. Depletion of EB1 or EB3 suppresses primary cilia formation. In addition, ectopic expression of a C-terminal domain of EB1 disrupts the localization of endogenous EB1 at the centrosomes and inhibits ciliogenesis, indicating a role for centrosomal EB1 in ciliogenesis.

### CEP104

CEP104 was initially identified as a centrosome protein and localizes to the mother and daughter centrioles but not to the basal body [22,23]. During ciliogenesis, CEP104 moves to the ciliary tip [24,25]. Its depletion or truncation mutation reduces the number of ciliated cells in mammalian cell lines [23,24,26] and in *Tetrahymena* [25] though the ciliary length is reduced in mammalian cells but unaffected in *Tetrahymena*. In *Chlamydomonas*, the function of CEP104/FAP256 in ciliogenesis has also been investigated [24]. Although the ciliary phenotype of the steady cells of the null mutant has not been described, a majority of mutant cells are unable to form cilia during cilia regeneration after deciliation and the cilia that do form exhibit a slower rate of assembly and are ca. 20% shorter than wild-type cilia at 180 min after deciliation. These data in combination with the data shown in mammalian cells and *Tetrahymena* indicate that CEP104 has a conserved role in the initiation of ciliary assembly. CEP104 localizes to the tip of axonemal MTs in *Chlamydomonas* and *Tetrahymena* and its loss modulates the relative length between central pair MTs and the doublet MTs, and that between A-tubules and the B-tubules at the tip of existing cilia [24,25,27]. These data suggest that CEP104 functions by regulating axonemal MT assembly.

CEP104 consists of a jelly-roll domain at the N-terminus, two coiled-coil domains and a TOG domain in the middle, a zinc finger domain and a SxIP motif at the C-terminus [28,29]. CEP104 interacts with CEP110 and CEP97 via the zinc finger domain and the jelly-roll domain, respectively, whereas interacts with EB1 via its SxIP motif [23,28,29]. One of the coiled-coil domains is involved in CEP104 dimer formation and the TOG domain binds tubulin [28,29]. It can be proposed that CEP104 functions as a dimer *in vivo* and promotes MT polymerization similar to Stu2 [30]. Indeed, the TOG domain of CEP104 has MT polymerizing activity *in vitro* and is essential for ciliary assembly. In contrast, the SxIP and the zinc finger domain are dispensable for ciliary assembly [31]. Thus, the role for CEP104 in ciliary assembly is independent from EB1 and CP110. Ciliogenesis is followed usually after removal of CP110 at the basal body or mother centriole. However, in a portion of CEP104 mutant cells, CP110 is not detectable at the basal body but no cilia are formed [26]. Given that CEP104 promotes MT polymerization *in vitro* [31], it indicates that CEP104 promotes axonemal MT assembly via its TOG domain after CP110 removal.

CEP104 may cooperate with CSPP1 in regulating axonemal MT dynamics because it interacts with CSPP1 shown by yeast two hybrid screening and proximity labeling [26,28], whereas CSPP1 promotes ciliary assembly (see below). In addition to regulating ciliogenesis, defects in CSPP1 and CEP104 also impair hedgehog signaling [26], which is consistent with the role of KIF7 (a member of kinesin-4 family, see later section) in modulating both ciliary tip MTs and hedgehog signaling [32,33]. Human CEP104 variants were shown to cause Joubert syndrome (JBTS), a recessive neurodevelopmental ciliopathy [34], which may be due to aberrant hedgehog signaling [35].

### CSPP1

CSPP1 was first identified as a centrosome/spindle pole associated protein and has two splicing forms: CSPP and CSPP-L [36,37]. CSPP1 localizes to the basal body and ciliary axoneme, and is shown to be involved in ciliogenesis [26,38]. Ectopic expression of CSPP-L increases ciliary length, whereas depletion of either isoforms results in shorter cilia and an increase in the number of aciliated cells [38]. CSPP-L appears to be the dominant form in ciliary function because its depletion results in similar phenotypes to depletion of both forms [38], which is supported by knockout of CSPP-L [26]. CSPP-L is enriched at the ciliary tip [26], suggesting that it may function in modulating MT dynamics at the ciliary tip. CSPP-L has been shown to be a MT lumen protein. It binds to and stabilizes growing MT ends *in vitro* [39] and cytoplasmic microtubules [40]. Similar to CEP104, mutations in CSPP1 also lead to JBTS [41–43]. CEP104 physically interacts with CSPP1 but they are mutually independent for ciliary localization. Double depletion for *CEP104* and *CSPP1* results in a more severe ciliary phenotype than depletion of CEP104 or CSPP1 alone, suggesting that they function independently [26].

### Crescerin

Crescerin is a class of TOG domain array-containing proteins identified by structure-based Blast searches using individual TOG domains from ch-TOG and CLASP family members [44]. Human Crescerin1 (FAM179B, TOGARAM1) has 4 TOG domains designated as TOG1-4 while Crescerin 2 (FAM179A) has two TOG domains similar to TOG3 and TOG4 of Crescerin1, respectively. *In vitro* analysis showed that TOG2 and TOG4 domains, but not TOG1 or TOG3 domains, increases the rate of MT polymerization. Interestingly, TOG3 and TOG4 domains are required for MT lattice association, whereas TOG1 and TOG2 are not. These data indicate that TOG3 and TOG4 domains bind MTs while TOG2 functions in stimulation MT polymerization [44].

CHE-12, a homologue of Crescerin1 in *C. elegans* localizes to the amphid and phasmid cilia [44,45]. In *che-12* mutant, the cilia are shorter and lack the distal segment [44,45], indicating that CHE-12 may regulate the A-tubule assembly. The *che-12* mutant without MT binding ability still localizes to cilia and profoundly reduces ciliary length [44], suggesting that CHE-12 mainly functions in tubulin binding to stimulate axonemal MT assembly. In *Tetrahymena*, knockout of its Crescerin homologue results in fewer cilia with slightly reduced length (16%). Interestingly, it was found at the plus ends of ciliary B-tubules and was thought to regulate the polymerization of the B-tubules more effectively [25], indicating that its role in *Tetrahymena* is distinct from that of CHE12 in regulating the A-tubules in worm [44,45].

In mammalian cells, TOGARAM1/Crescerin1 localizes to cilia as well and is enriched at the ciliary tip and basal body when overexpressed [44]. Its mutations result in short cilia and JBTS [46,47]. Because the short cilia are associated with decreased axonemal acetylation and polyglutamylation and undergo disassembly upon cold treatment, it is proposed that TOGARAM1 functions in stabilizing axonemal MTs [46].

In *Chlamydomonas,* SHF1, the only homologue of Crescerin1*,* contains TOG2, TOG3, and TOG4 domains, and loss-of-function mutations in *SHF1* result in short cilia [48,49]. SHF1 is present as puncta along the cilium and enriched at the ciliary tip. During ciliary regeneration of *shf1*, the initial assembly rate of cilia is unaffected, suggesting that SHF1 may not be directly involved in axonemal MT polymerization at the ciliary tip. It is proposed that SHF1 loads tubulins in the cytoplasm and transports them to the ciliary tip for axonemal assembly [48]. However, SHF1 may also have a direct role in polymerization of axonemal MTs. Its function may depend on the local tubulin concentration at the ciliary tip. Along with the elongation of cilia during ciliary assembly, the delivery of tubulin at the ciliary tip gradually decreases [50–52]. When tubulin levels fall below a certain threshold that correlates with a specific ciliary length, SHF1 may be required to capture tubulins via its TOG domain, analogous to Stu2 [30], to facilitate proper MT assembly. Consequently, the loss of SHF1 would impact ciliary assembly above a minimal length, which could explain the *shf1* phenotype with shorter ciliary length and normal rate of ciliary assembly during the early stages of ciliogenesis.

### ARMC9

ARMC9 was initially identified to be mutated in JBTS [53]. It localizes to the daughter centriole and basal bodies in mammalian cells. Genetic inactivation of ARMC9 in zebrafish results in defects in ciliogenesis including curved body and decreased cilia number. ARMC9 is a protein of 818 aa and predicted to have a N-terminal LisH domain, a coiled-coil domain and an armadillo repeats domain in the middle [53]. The LisH domain is suggested to regulate MT dynamics likely through binding to MTs [54]. In patient fibroblasts carrying ARMC9 variant, the ciliary length is significantly reduced, which is consistent with shortened cilia phenotype in ARMC9 knockout cells in zebrafish [46]. It was further shown that ARMC9 functions in regulating ciliary stability similarly to TOGARAM1. ARMC9 directly interacts with TOGARAM1 and CSPP1. Thus, it indicates that ARMC9, TOGARAM1, and CSPP1 can form a functional module to regulate ciliogenesis, which is supported by the fact that the human variants of all the three proteins cause JBTS (see above). It was also found that ARMC9 physically associates with CCDC66, which localizes to the basal body, the axoneme and ciliary tip of primary cilia. Depletion of CCDC66 results in shorter cilia and a lower rate of ciliated cells [55,56]. In mouse or dog, its mutation causes retinal degeneration [57,58]. CCDC66 is a coiled-coil domain containing protein without known MT binding motifs or domains. It may not directly regulate axonemal MT dynamics.

In *Tetrahymena*, both ARMC9A and its paralog localize to the ciliary tip while ARMC9A is enriched at the ends of B-tubule [25]. In contrast to the shortened ciliary phenotype observed in ARMC9 human variants or zebrafish knockout [46], *ARMC9A* null mutation slightly increases ciliary length (14%). It was also found that the B-tubules are longer in relation to the A-tubules, suggesting that the A- and B-tubules may be differentially regulated by ARMC9 in this organism [25]. Although the effects on ciliary length of ARMC9s in *Tetrahymena* and vertebrates are inconsistent, ARMC9 in vertebrates also appears to function at the ciliary tip. It has been shown that ARMC9 in mammalian cells directly interacts with TOGARAM1 and CSPP1, which are enriched at the ciliary tip [26,46].

### MAP9

MAP9 was initially found to be associated with cytoplasmic MTs and directly interacts with MTs via its C-terminal region that contains a microtubule interacting and transport (MIT)-like domain [59,60]. It is associated only with doublet MTs but not singlet MTs in cilia of *C. elegans* and mammalian cells. Loss of MAP9 in *C. elegans* impairs the formation and stability of the doublet MTs as well as axonemal motor activities [61]. A function of MAP9 has also been found in primary and photoreceptor cilia [62].

### RP1 and RP1L1

Retinitis pigmentosa 1 (RP1) is specifically expressed in retina and its mutation causes retinitis pigmentosa, a group of eye diseases that lead to loss of vision [63,64]. RP1 is localized to the connecting cilium and axoneme in the outer segment of the photoreceptor cells [65,66]. Partial deletion mutants of RP1 in mice exhibit shorter photoreceptor axonemes. RP1 interacts with MTs via the doublecortin domain and promotes MT polymerization *in vitro*. Cytoplasmic MTs are resistant to nocodazole-induced disassembly in RP1 overexpressed mammalian cell lines [66]. These data suggest that RP1 is a MT stabilizer and function in stabilization of photoreceptor axonemes. RP1-like 1 (RP1L1), which also contains the doublecortin domain [67,68], localizes to the photoreceptor axonemes as well. Loss of RP1L1 in mice causes photoreceptor degeneration. RP1 and RP1L1 interact with each other and synergistically regulate the stability of the photoreceptor axonemes [69]. Because RP1 expression is only restricted in retina but not other tissues [65], it would not be involved in primary cilia formation.

**Figure 1:**
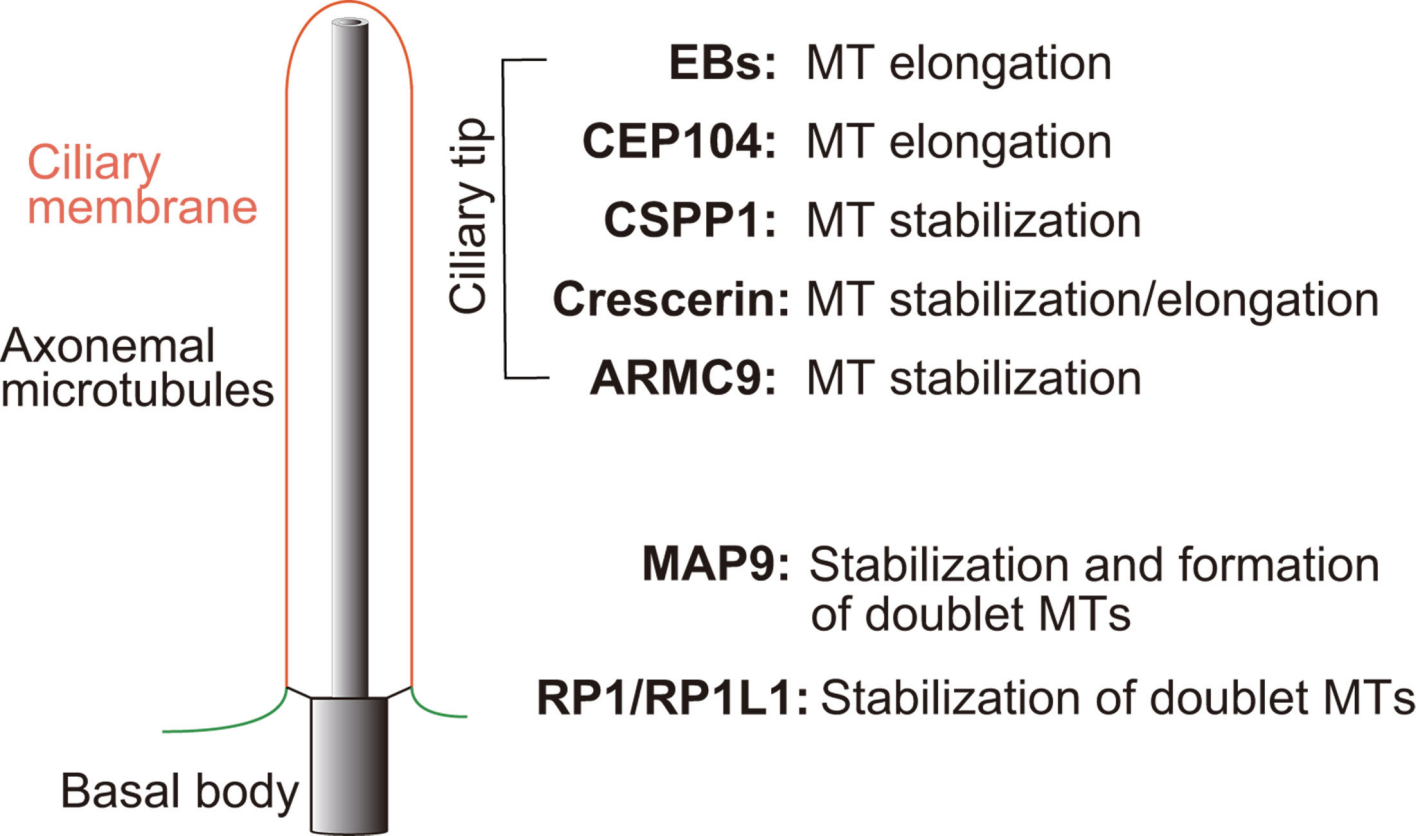
Regulation of axonemal microtubules by MT binding proteins. Shown on the left is a simplified structure of a cilium, which consists of a basal body, a transition zone, an axonemal microtubule core and ciliary membrane. The ciliary membrane is connected with the cytoplasmic membrane (green). The functions of microtubule-binding proteins on modulation of axonemal microtubules are shown on the right. Please see text for details.

The working mechanisms of the above-mentioned microtubule binding proteins in regulating axonemal microtubules are depicted in Figure 1.

## Regulation of axonemal MT dynamics by kinesins

**Figure 2:**
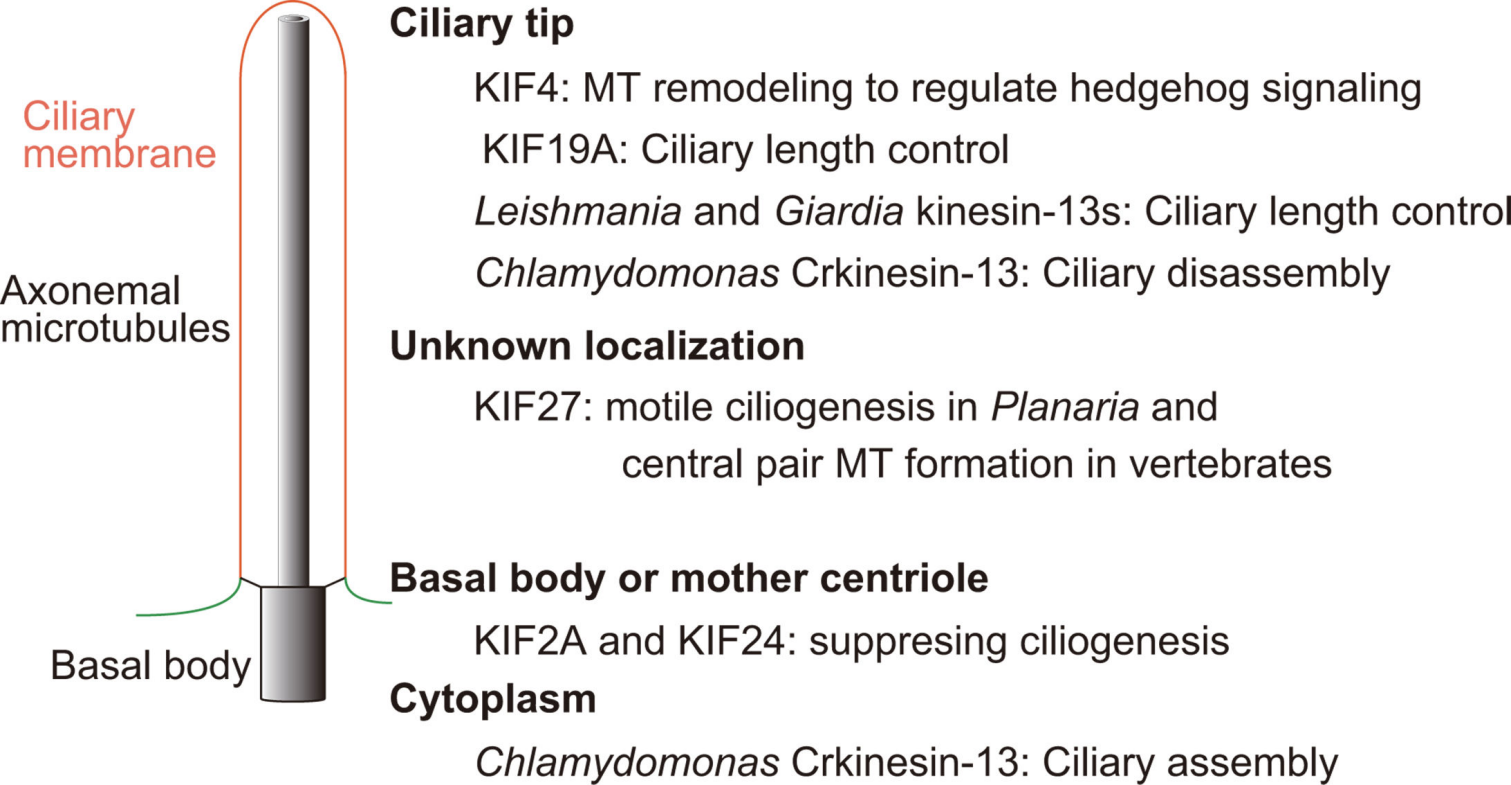
Regulation of ciliary assembly and/or disassembly by kinesins. Shown on the left is a simplified structure of a cilium (see legend of Figure 1). The functions of kinesins in regulating ciliary assembly and/or disassembly are shown on the right. Please see text for details.

Based on sequence homology, kinesins are classified into 15 families [70], most of which are best known for their functions in intracellular transport [71]. However, some members of the kinesin-4, kinesin-8, and kinesin-13 family exhibit functions in modulating MT dynamics [13,72,73]. It has been demonstrated that all these kinesins are also involved in ciliary assembly and/or disassembly. A diagram depicting the role of these kinesins in regulating ciliary assembly and/or disassembly is shown in Figure 2. Please see below for details.

### Kinesin-4 family

KIF7, a member of the kinesin-4 family, has been found to localize to the tip of primary cilia. Genetic inactivation of *KIF7* in cultured fibroblasts results in slightly longer and less stable cilia [32,33]. KIF7 lacks MT-dependent motility *in vitro* [32,74]. Instead, it binds preferentially to the GTP-bound MT plus ends, and decreases the rate of MT polymerization and induces MT catastrophe, leading to shorter MTs [32]. Because KIF7 binds to GLI proteins, core effectors of the hedgehog signal pathway, the major function for KIF7 appears to regulate the hedgehog signaling at the ciliary tip by tethering signaling molecules [13,32].

KIF27, a closely related member of KIF7 in the kinesin-4 family, has been implicated in motile cilia assembly. RNAi depletion of Smed-kif27, a planarian homologue of KIF7/KIF27, abolishes motile cilia assembly in *Schmidtea mediterranea* [75]. Mouse KIF27 can ectopically interact with Fu/STK36, whose null mutation results in aberrant structures of motile cilia with two-thirds lacking central pair MTs. Furthermore, depletion of a homologue of KIF27/KIF7 in zebrafish that interacts with Fu results in disruption of left–right asymmetry, indicating defects in motile cilia formation [76]. These data indicate that KIF27 and its functional homologues in vertebrates participate in motile ciliogenesis. However, it is not clear how KIF27 exerts its function regarding ciliogenesis. A synthetic KIF27 motor dimer possesses slow and processive motility *in vitro* [74], it remains to be shown whether it has activity in influencing MT dynamics. Interestingly, in contrast to KIF7 and KIF27, KIF4 and KIF21, the other members of kinesin-4, are fast and processive motors [74], indicating that the sequence difference between different members of the same family can profoundly affect the motor behaviors of a kinesin.

### Kinesin-8 family

Kinesin-8 family members are MT plus end-directed motors that exhibit MT depolymerizing activity at the plus end [72]. Among the four members of this family including KIF18A, 18B, 19A and 19B, KIF19A is implicated in ciliary length control of motile cilia in mouse [77]. It localizes to the ciliary tips of motile cilia. Knockout of KIF19A results in longer motile cilia, which is supported by another study [78]. However, the length of primary cilia is not affected in KIF19A knockout mice [77]. In *C. elegans*, mutations of KLP-13, a homologue of KIF19A, result in longer cilia [79]. Furthermore, KLP-13 mutations can suppress degeneration of axonemal MTs in *nekl-4*(PESTΔ) mutants [80], indicating that KIF19A and its homologues can modulate axonemal MT dynamics regardless of the presence or absence of the central pair MTs. The divergent effects of KIF19 are thus cell type and organism-specific, which may depend on differential regulation of gene expressions.

### Kinesin-13 family

KIF2C or MCAK, the founding member of kinesin-13 family that also includes KIF2A, KIF2B and KIF24, is immotile and can be targeted to both ends of MTs by diffusion to depolymerize MTs [81,82]. Studies in unicellular organisms and mammalian cells showed that kinesin-13s are involved in different aspects of ciliogenesis including ciliary assembly, length control and ciliary disassembly.

### Regulation of ciliary assembly, length and disassembly by kinesin-13 members in unicellular organisms

In *Leishmania*, LmjKin13-2, a member of the kinesin-13 family was mainly found in flagella with enrichment at the flagellar tip. Its overexpression results in shorter flagella, whereas its depletion produces longer flagella [83]. This role in controlling ciliary length is supported by studies in *Giardia* [84]. In contrast, the homologue of LmjKin13-2 in *Trypanosome* has a marginal impact on flagellar assembly [85]. This result might be owing to redundant functions of multiple members of kinesin-13 in this organism. CrKinesin-13, the single member of kinesin-13 in *Chlamydomonas*, regulates both ciliary assembly and disassembly. CrKinesin-13 is transported to cilia upon induction of ciliary disassembly and positively regulates ciliary disassembly [86]. Since ciliary disassembly occurs at the ciliary tip [87], Crkinesin-13 presumably depolymerizes axonemal MTs from the plus ends at the ciliary tip. However, depletion of CrKinesin-13 does not induce longer cilia instead of shorter cilia. It has been found that depletion of CrKinesin-13 prevents depolymerization of cytoplasmic MTs and, thus, would impair the delivery of cytoplasmic tubulins for ciliary assembly [88]. Taken together, the regulation of cilia by the members of kinesin-13 may be cell type-specific.

### Suppressing ciliogenesis by KIF2A in mammalian cells

In mammalian cells, KIF2A plays a critical role in ciliary dynamics, whereas KIF2B has a minor role and KIF2C is dispensable for cilia function [89]. KIF2A localizes to the subdistal appendages of the mother centriole or the basal foot of the basal body. Its overexpression reduces the number of ciliated cells in serum-starved quiescent RPE1 cells. In contrast, knockout of KIF2A does not alter ciliation rate and ciliary length, indicating that KIF2A is not required for ciliary assembly or length control but instead can suppress ciliogenesis. KIF2A activity can be regulated by PLK1 mediated protein phosphorylation at T554. This phosphorylation enhances MT depolymerizing activity as shown *in vitro*. Constitutive activation of PLK1 reduces ciliogenesis. The data support a model in which KIF2A functions in suppressing ciliogenesis in proliferating cells.

The function of KIF2A in suppressing ciliogenesis is corroborated by the following studies [90,91]. WDR62 interacts with and recruits CEP170 to the basal body, while CEP170 further recruits KIF2A [90]. Knockout of WDR62 leads to increased ciliation and ciliary length in neuronal progenitor cells in cerebral organoids and in mice. Overexpression of KIF2A rescues the ciliary phenotypes in WDR62^−/−^ cells, indicating that WDR62 exerts its function via KIF2A to suppress ciliogenesis. In HEK293 cells, overexpression of KIF2A suppresses ciliogenesis whereas its knockout increases the percentage of ciliated cells [91]. Thus, all the current data support a role for KIF2A in suppressing ciliogenesis. However, how KIF2A suppresses ciliogenesis requires further investigation. It may depolymerize cytoplasmic MTs surrounding the mother centriole to prevent delivery of ciliary precursors needed for cilia assembly [92].

Conflicting data exist for an additional role of KIF2A in ciliary shortening or disassembly.

Upon serum stimulation to serum-starved RPE-1 cells, ciliary shortening is delayed in KIF2A knockout cells, suggesting that KIF2A participates in ciliary shortening as well [89]. As discussed above, WDR62 recruits KIF2A to the basal body. However, upon serum stimulation of serum starved WDR62^−/−^ MEF cells, the rate of ciliary shortening is similar to that of the wild-type cells [90], indicating that KIF2A is not involved in ciliary disassembly. Thus, these conflicting data may be related to different cell lines used.

### Suppression of ciliogenesis by KIF24

KIF24, another member of the kinesin-13 family, has been found to regulate ciliogenesis in mammalian cells [93]. It preferentially localizes to the distal end of the mother centriole. Its loss from cycling cells induces aberrant ciliary assembly, which can be explained by concomitant loss of CP110, a negative regulator of ciliary assembly [94]. Thus, KIF24 may be tethered to CP110 and other regulators to prevent ciliogenesis. On the other hand, KIF24 shows MT depolymerization activity *in vitro*. It binds and remodels centriolar MTs. How could its MT disassembly activity control ciliary assembly? When the mother centriole is converted to basal body, CP110-CEP97 is removed at the distal end of the mother centriole to allow transition zone formation [5,94]. The transition zone has nine outer doublet MTs that are extended from the A and B tubules of the basal body triplet MTs. It could be hypothesized that KIF24 functions to suppress transition zone MT assembly. A reduction in its activity or alteration in its localization, as a result, may enable the assembly of transition zone at the initiation of ciliogenesis. Similar to KIF2A, the MT depolymerizing activity of KIF24 is regulated by phosphorylation [89]. Phosphorylation of KIF2A by Nek2 prevents ciliary assembly in cycling cells.

The regulation of ciliogenesis by members of kinesin-13 is complex. It is involved in suppressing ciliogenesis in proliferating cells, regulation of ciliary length and assembly and promoting ciliary shortening or disassembly. This functional heterogeneity may depend on the unique properties of each kinesin member, gene redundancy, precise cellular localization, distinct cellular context, and likely different modes of regulation.

**Perspectives**

- *Importance of the field*: Cilia are present in almost every cell in the human body. Their functions in cell motility and signaling are tightly associated with development and physiology. Defects in cilia formation, structure and cilia-based signaling are linked with a cohort of human diseases, termed ciliopathies. The backbones of cilia are axonemal MTs. Thus, axonemal MT dynamics can affect ciliary assembly, disassembly and signaling as well. Elucidating the underlying mechanisms of axonemal MT dynamics not only deepens our understanding on ciliogenesis but also provides insights into MT dynamics as a whole. In addition, a number of human variants that cause ciliopathies are characterized by their dysfunctions in regulating axonemal MT dynamics, which underscores medical significance.
- *Current thinking*: The dynamic regulation of cytoplasmic MTs has been under intensive study in the past several decades. In contrast, control of ciliary MTs appears to be an emerging field. In particular, the assembly and disassembly of the doublet MTs may shed new light on MT dynamics within a ciliary context. The assembly of A- and B-tubules is tightly coupled. In addition, the assembly of the nine outer doublet MTs and/or central pair MTs are also coordinated. Post-translational modifications and the presence of numerous MT inner proteins may influence the dynamics of axonemal MTs in a way different from those in cytoplasmic MTs. Though cilia are ancient organelles, the mechanisms that regulate in axonemal MT dynamics appear to be organismal- and cell type-specific. Gene duplications and losses, differential gene expression, gene redundancy, the slight divergence in amino acid sequence of protein homologues, as well as the presence of specific regulators would contribute to different modes of regulations for ciliary dynamics.
- *Future directions*: Studies on cytoplasmic MTs have identified numerous effectors that are tightly associated with the regulation of MT dynamics. However, only a few of them have been studied for their function in cilia. It is expected that other modulators of cytoplasmic MT dynamics may also function in cilia. The doublet MTs in cilia that differ from singlet cytoplasmic MTs may be regulated by novel and uncharacterized regulators. Many protein kinases have been found to regulate ciliary assembly and/or disassembly [95–99], but the molecular substrates for these kinases remain essentially unknown. The identified and/or to-be molecules in regulating axonemal MT dynamics may potentially be substrates of these cilia-associated kinases. The properties of axonemal MT dynamics and modulation by its effectors will require a joint effort, including but not limited to genetic, biochemical, and cell biological including live imaging approaches.

## Open Access

This article has been published open access under our Subscribe to Open programme, made possible through the support of our subscribing institutions, learn more: here https://portlandpress.com/pages/open_access_options_and_prices#conditional

## Abbreviations

JBTS: Joubert syndrome
MT: Microtubules
RP1: Retinitis pigmentosa 1
RP1L1: RP1-like 1
